# The Relationships between Mental Health Symptoms and Gambling Behavior in the Transition from Adolescence to Emerging Adulthood

**DOI:** 10.3389/fpsyg.2017.00478

**Published:** 2017-03-31

**Authors:** Dominic Sagoe, Ståle Pallesen, Daniel Hanss, Tony Leino, Helge Molde, Rune A. Mentzoni, Torbjørn Torsheim

**Affiliations:** ^1^Department of Psychosocial Science, University of BergenBergen, Norway; ^2^Department of Social and Cultural Sciences and Social Work, Darmstadt University of Applied SciencesDarmstadt, Germany; ^3^Department of Clinical Psychology, University of BergenBergen, Norway; ^4^KoRus-Øst, Innlandet Hospital TrustOttestad, Norway

**Keywords:** adolescence, emerging adulthood, gambling, latent class analysis, longitudinal, mental health, youth

## Abstract

There is a paucity of longitudinal investigations of gambling behavior in the transition from adolescence to emerging adulthood. We conducted a longitudinal investigation of the associations and patterns of change between mental health symptoms and gambling behavior. A representative sample of Norwegians completed questionnaires containing demographic, mental health, and gambling measures at age 17 (*N* = 2055), and at ages 18 (*N* = 1334) and 19 (*N* = 1277). Using latent class analysis, three classes of gambling behavior were identified: consistent non-gambling (71.1%), consistent non-risk gambling (23.8%), and risky-and-problem gambling (5.1%). Being male, showing higher physical and verbal aggression and having more symptoms of depression were associated with greater odds of belonging to the risky-and-problem gambling class at age 17. Overall, the risky-and-problem gambling class had the highest physical and verbal aggression, anxiety, and depression at 19 years. Our findings elucidate the reciprocal relationship between mental health and gambling behavior in the transition from adolescence to emerging adulthood, and the importance of recognizing these factors in designing targeted interventions.

## Introduction

Gambling problems have been associated with various disorders including substance abuse, depression, anxiety disorder, attention-deficit hyperactivity disorder, and suicide (Raisamo et al., 2013; Cook et al., 2014; Black et al., 2015; Chinneck et al., 2016). Also, gambling in adolescence has been linked to more severe gambling-related problems later in life (Burge et al., 2004; Lynch et al., 2004). Problem behavior theory (PBT; Jessor and Jessor, 1977) suggests a connection between dysfunctional behaviors where involvement in one problem behavior (e.g., underage gambling) is linked to involvement in other problem behaviors (e.g., substance abuse). In support of PBT, gambling has been positively associated with a spectrum of addictive syndromes such as exercise, Internet, and work addiction in adolescence and emerging adulthood (Villella et al., 2011). Indeed, gambling has been identified as symptomatic of a tendency toward risky behavior in general (Petry, 2000; Mishra et al., 2010).

In terms of gambling problems, ‘stability’ refers to the propensity for individuals to remain at one diagnostic level rather than oscillating between recovery and deterioration (LaPlante et al., 2008). Evidence from a systematic review of five longitudinal studies indicates that gambling behavior and problems are unstable and multidirectional (LaPlante et al., 2008). Also, higher aggression among females and lower aggression among males in early adolescence have been associated with higher odds of risky gambling in late adolescence (Yücel et al., 2015). There is additional evidence that being at risk for problem gambling generally diminishes with time (Edgerton et al., 2015). Moreover, temperament and high-risk behaviors in early adolescence have been found to predict higher odds of risky gambling in late adolescence. Further, a reciprocal relationship has been established between aggression and gambling over time, after controlling for previous levels of aggression and gambling (Adachi and Willoughby, 2013). It can be inferred from the above that the relationship between risky behavior as well as mental health symptoms and gambling problems is unstable, multidirectional, and reciprocal over time.

Hence, longitudinal studies are important in the effort to better understand the associations between gambling behavior and mental health symptoms (Kong et al., 2014; Carbonneau et al., 2015). However, there is a lack of longitudinal research on gambling behavior in the transition from adolescence to emerging adulthood (Dussault et al., 2011; Scholes-Balog et al., 2014; Yücel et al., 2015). One limitation of previous longitudinal investigations (Adachi and Willoughby, 2013; Edgerton et al., 2015; Yücel et al., 2015; Chinneck et al., 2016) is that they have not been based on representative samples. Accordingly, the external validity of findings from these studies is limited. Thus, the prospective association between gambling involvement and mental health symptoms in the transition from adolescence to emerging adulthood remains unclear.

The self-medication model of addiction (Khantzian, 1985) suggests that individuals engage in addictive behaviors as self-medication or as a means to experiencing relief from debilitating affective conditions. In line with the self-medication model, mental health symptoms associated with or co-occurring with gambling problems among youth include aggression (Adachi and Willoughby, 2013), anxiety (Ste-Marie et al., 2006), depression (Dussault et al., 2011), and loneliness (Hing et al., 2015). However, the extensive use of cross-sectional designs makes it difficult to make conclusions about the directionality or ‘stability’ of the association between gambling behavior and mental health symptoms (Langhinrichsen-Rohling, 2004). In order to further investigate the relationship between gambling behavior and mental health in the transition from adolescence to emerging adulthood (from age 17 to 19), we conducted a latent class analysis of the associations and patterns of change among demographic variables (sex, grade point average, and parental education), mental health factors (loneliness, physical aggression, verbal aggression, anxiety, and depression) and gambling behavior.

## Materials and Methods

### Sample

A total of 3000 17-year-olds (females = 50%) were randomly selected from the Norwegian National Population Registry and invited to participate in a survey about gambling behavior in 2012. Due to invalid addresses, 54 individuals could not be reached and were therefore excluded. In addition, parents and guardians of 23 other individuals informed us that their offspring were unable to participate for reasons such as intellectual disability. A total of 2059 of the 3000 contacted individuals completed and returned the questionnaire (response rate = 70.4%, i.e., 2059 of 2923). We excluded four respondents, as they were younger than 17 years. The final sample therefore comprised 2055 17-year-olds (females = 53.0%). Four participants did not indicate their sex. Those who responded in 2012 were contacted again in 2013 (aged 18 years) for the second wave. In the second wave, a total of 1334 individuals responded (females = 58.7%, retention rate = 64.9%). In the third wave, respondents to the first wave were contacted again in 2014 (aged 19 years). In total, 1277 individuals responded (females = 61.7%) resulting in a retention rate of 62.1%. Other characteristics of the sample are presented in **Table 1**.

**Table 1:** Characteristics of the study sample at ages 17 and 19.

		Age 17			Age 19		
			
		(*N* = 2055)			(*N* = 1277)		
			
	Range	*n*	*M*	*SD*	*n*	*M*	*SD*
Loneliness^a^	0–3	2001	0.61	0.49	1244	0.64	0.54
Physical aggression^b^	0–4	2031	0.70	0.86	1269	0.46	0.70
Verbal aggression^b^	0–4	2030	1.09	0.79	1272	0.93	0.71
Anxiety^c^	0–21	2021	5.58	3.52	1253	5.34	3.70
Depression^c^	0–21	2021	3.68	2.98	1260	3.04	3.05


*^a^Robert’s UCLA Loneliness Scale [*never* (0) to *often* (3)]; ^b^Short-Form Buss-Perry Aggression Questionnaire [*very unlike me* (0) to *very like me* (4)]; ^c^Hospital Anxiety and Depression Scale [scored from 0 (e.g., *not at all*) to 3 (e.g., *very often indeed*) with higher scores indicating higher symptoms].*

### Measures

A self-report questionnaire was used in the survey. For each wave, the questionnaire contained the following measures.

#### Demographics

Demographic variables assessed in the survey questionnaire included age, sex, grade point average, and parental education.

#### Gambling Behavior and Problems

The Problem Gambling Severity Index (PGSI), a subscale of the Canadian Problem Gambling Index (CPGI; Ferris and Wynne, 2001) was used in the assessment of gambling behavior and problems. Although originally developed for use with adults, the PGSI has been used in studies of adolescents (Emond et al., 2011) and populations including both adolescents and adults (Huang and Boyer, 2007; Wardle et al., 2011; Currie et al., 2013). Accordingly, we preferred the PGSI as we longitudinally surveyed participants in adolescence and young adulthood. The PGSI consists of nine-items (e.g., “Have you borrowed money or sold anything to get money to gamble?”) addressing gambling behavior and problems. All items are answered on a four-point rating scale ranging from “never” (0) to “almost always” (3). Responses to each wave were based on experiences from the preceding 12 months (past year).

An index score was computed for each wave by summing scores across the nine-items. Higher scores on the PGSI indicate greater propensity to problem gambling. In line with proposed categorization (Ferris and Wynne, 2001), based on index scores, participants were initially assigned to one of five categories: non-gambling (no gambling during the last 12 months), non-problem gambling (index score = 0), low risk gambling (index score = 1 or 2), moderate risk gambling (index score = 3 to 7), and problem gambling (index score ≥ 8). Cronbach’s alpha were 0.85 for wave 1, 0.94 for wave 2, and 0.83 for wave 3.

#### Loneliness

Robert’s UCLA Loneliness Scale (RULS-8; Roberts et al., 1993) was used for assessing loneliness. This eight-items instrument is answered along a four-point Likert-type scale ranging from “never” (0) to “often” (3). An example item is “I lack companionship.” Four-items were reverse-coded. No specific time frame in terms of experiencing loneliness was given. A composite score was computed by summing responses across all items. Index scores were computed for each wave. RULS-8 yielded Cronbach’s alpha of 0.76, 0.81, and 0.80 for waves 1, 2, and 3, respectively.

#### Aggression

The physical and verbal aggression subscales of the Short-Form Buss-Perry Aggression Questionnaire (BPAQ-SF; Diamond and Magaletta, 2006) were used in the assessment of aggression. The physical subscale consists of four-items (e.g., “Given enough provocation, I may hit another person”) with the verbal subscale containing three-items (e.g., “I often find myself disagreeing with people”). Items are answered on a five-point scale ranging from “very unlike me” (0) to “very like me” (4), with higher scores on each subscale denoting higher inclination to physical and verbal aggression respectively. No specific time frame in terms aggressive behavior was provided. For each wave, an index score was computed for each subscale by summing responses across all items. Cronbach’s alpha for the physical subscale were 0.80, 0.76, and 0.78 for waves 1, 2, and 3, respectively. For the verbal subscale, Cronbach’s alpha were 0.66 for wave 1, 0.68 for wave 2, and 0.67 for wave 3.

#### Anxiety and Depression

The Hospital Anxiety and Depression Scale (HADS; Zigmond and Snaith, 1983) was used in the assessment of symptoms of anxiety and depression. HADS consists of an anxiety subscale (seven-items, e.g., “I get sudden feelings of panic”) and a depression subscale (seven-items, e.g., “Worrying thoughts go through my mind”). Items are answered on a four-point scale scored from 0 (e.g., “not at all”) to 3 (e.g., “very often indeed”). Three-items were reverse-scored for each subscale. Responses for each wave were based on past week experiences. An index score, per wave, was computed for each subscale by summing responses across corresponding items with higher scores on each subscale signifying greater symptoms of anxiety or depression. Cronbach’s alpha for the anxiety subscale were 0.76 for wave 1, 0.80 for wave 2, and 0.81 for wave 3. The depression subscale yielded Cronbach’s alpha of 0.69 at wave 1, 0.73 at wave 2, and 0.76 at wave 3.

### Procedure

Participants were randomly selected from the Norwegian National Registry in 2012. They were sent an invitation package by postal mail and invited to participate in the study. An invitation letter described the purpose of the study and indicated that researchers at the University of Bergen, Norway were conducting the study. The letter also assured participants that the information they provided would be kept confidential, and mentioned that all participants would receive a gift card worth NOK 200 (≈ US$ 30) as compensation for participating in the study. The questionnaire package was sent out approximately 1 week after the invitation letter was sent. It included: (a) an introductory letter with sections on confidentiality and informed consent, (b) the questionnaire, (c) an instruction on how to complete the questionnaire, and (d) a pre-paid envelope for returning the questionnaire. Participants had the option to complete either the paper version of the questionnaire or an online version. A web link to the online version was provided in the instruction document. A maximum of two reminders were sent by postal mail to those who had not replied. This procedure was repeated in 2013 and 2014 for the second and third waves respectively. The study was carried out in line with the Declaration of Helsinki. All participants provided written informed consent and the study received ethical approval from the Regional Committee for Medical and Health Research Ethics in South East Norway (project number: 2012/914).

### Statistical Analysis

For attrition analysis, we constructed a nominal missingness variable using the following categorization: 1 = no missing; 2 = missing on wave 2 only; 3 = missing on wave 3 only; 4 = missing on both waves 2 and 3. We conducted a multinomial regression of missingness using the wave 1 demographic (sex, grade point average, father’s and mother’s education) and mental health (loneliness, gambling, physical and verbal aggression, anxiety, and depression) variables as predictors. The no missing group served as reference category for missingness.

Descriptive statistics were used to ascertain characteristics of the sample. Latent class analysis (LCA; Collins and Lanza, 2010) was conducted to empirically examine quantitative patterns of change and stability in the relationship between mental health factors and gambling behavior across the three waves (from ages 17 to 19 years). In the context of the present study, LCA postulates the existence of an unobserved latent class variable with discrete different underlying patterns of gambling across measurements.

The categorical classification of problem gambling (Ferris and Wynne, 2001) was used as observed variables in the computation. The frequency of risky and problem gambling in our sample was very low. In order to avoid computational problems with sparse data, the three lowest frequency categories (low risk, moderate risk, and problem gambling) were collapsed. The latent class was therefore conducted on a three-category variable differentiating between non-gambling, non-risk gambling, and risky-and-problem gambling classes at each of the three time points. This method is consistent with previous analyses that merged different categories of the PGSI to increase statistical power (Svensson and Romild, 2014; Kairouz et al., 2015; Cowlishaw et al., 2016). A lack of support for a missing completely at random (MCAR) mechanism indicated that a listwise deletion of cases would potentially bias results. We therefore used all available data for the estimation of classes. This entailed using covariates as auxiliary data in the assessment, and as active predictors in classifying latent class membership.

To detect mental health precursors and endpoints of gambling classes, covariates measured at waves 1 and 2 were entered into the analysis. In the first set of analyses, examining the role of background factors on latent class membership, we regressed the best-fitting latent class solution on wave 1 demographic (sex, grade point average, father’s and mother’s education) and mental health (loneliness, physical and verbal aggression, anxiety, and depression) covariates. Multinomial regression splits a *k*-class dependent variable into *k-1* sets of regression equations. One of the *k* categories is used as reference for the odds of each of the *k-1* remaining categories. The non-gambling category was used as a reference for the other two categories. Hence, the first multinomial model compared the non-risk gambling class to the non-gambling class, whereas the second multinomial model compared the risky-and-problem gambling and the non-gambling classes. The Mplus R3STEP method (Asparouhov and Muthén, 2014) was used to avoid that covariates influenced the estimation of the number of latent gambling classes. The R3STEP method first estimates the number of latent classes. Next, it estimates the level of classification uncertainty. Finally, it regresses the latent class solution on covariates with information on latent class measurement errors.

In the final set of analyses, we examined endpoints of gambling class at wave 3 by regressing wave 3 mental health (loneliness, physical aggression, verbal aggression, anxiety and depression) endpoints on the best-fitting latent class solution, adjusting for sex. A modified procedure (Bolck et al., 2004) using the BCH method was used in Mplus (version 7.3). The BCH method can be used when latent class effect on distal endpoints is estimated, and there is a need to control for other covariates. It uses information on classification error by including classification weights for the estimated regression of endpoints on latent class and other covariates. The selection of number of classes was based on Bayesian information criterion (BIC; lower-is-better), entropy [values nearing 1 indicating clear separation of classes, i.e., higher-is-better (Celeux and Soromenho, 1996)], and the bootstrapped likelihood ratio test (BLRT; McLachlan and Peel, 2000) for comparing a model with *k* and *k-1* classes. In the current study, we estimated solutions with one to four classes.

## Results

### Sample Attrition

Of the 2055 participants in the first wave, 258 were missing on wave 2 only, 312 were missing on wave 3 only, and 463 were missing on both waves 2 and 3. In general, the association between wave 1 predictors and missingness were weak with the exception of sex. Father’s education level was negatively associated with dropout at wave 2 (OR = 0.81, *p* = 0.007) whereas being female was associated with lower odds of dropout at wave 3 (OR = 0.51, *p* = 0.000). Additionally, being female (OR = 0.32, *p* = 0.001), having higher grades (OR = 0.70, *p* = 0.001), and higher loneliness (OR = 0.71, *p* = 0.018) predicted lower odds of dropout at waves 2 and 3. Gambling, anxiety, depression, as well as physical and verbal aggression did not significantly predict missingness. The significant prediction of missingness was in line with a missing at random (MAR), albeit not a MCAR mechanism.

### Gambling Prevalence

The majority of respondents belonged to the non-gambling group, followed by the non-problem, low risk, moderate risk, and problem gambling groups. Age 17 had the highest prevalence of non-gambling (73.9%) while age 18 had the highest prevalence of non-problem gambling (29.2%) albeit slightly higher than age 19 (28.6%). Additionally, prevalence of low risk gambling was highest at age 19 (8.9%). Moderate risk gambling prevalence was similar for ages 18 (2.2%) and 19 (2.3%). Further, age 18 had the highest prevalence of problem gambling (0.8%). See **Table 2**.

**Table 2:** Prevalence of gambling at ages 17, 18, and 19.

		Age 17		Age 18		Age 19	
				
		(*N* = 2055)		(*N* = 1334)		(*N* = 1277)	
				
Category§	Index score	*n*	%	*n*	%	*n*	%
No gambling	-	1513	73.9	802	61.2	757	59.9
Non-problem gambling	0	416	20.3	382	29.2	361	28.6
Low risk gambling	1 or 2	83	4.1	86	6.6	112	8.9
Moderate risk gambling	3 to 7	30	1.5	29	2.2	29	2.3
Problem gambling	≥8	5	0.2	11	0.8	4	0.3


*Frequencies may not add up due to missing values.*

*§Problem Gambling Severity Index [*never* (0) to *almost always* (3)].*

### Model Fit

**Table 3** shows the model summary for the LCA estimated for one to four latent classes. The three-class solution was the best model as evident in lowest BIC (7328.42), highest entropy (0.61) and BLRT (*p* < 0.0001).

**Table 3:** Model summary for the latent class analysis.

Class	Parameters	LL	AIC	BIC	aBIC	Entropy	BLRT *p* <
One-class	6	-3769.70	7551.39	7585.15	7566.09	NA	NA
Two-class	13	-3625.30	7276.59	7349.75	7308.44	0.52	0.0001
Three-class	20	-3587.94	7215.88	7328.42	7264.88	0.61	0.0001
Four-class	27	-3583.80	7221.59	7373.52	7287.74	0.49	0.1622


*LL, Log likelihood; AIC, Akaike’s information criterion; BIC, Bayesian information criterion; aBIC, adjusted Bayesian information criterion; BLRT, Bootstrapped likelihood ratio test; NA, Not available.*

### Patterns of Gambling Behavior from Age 17 to 19

**FIGURE 1:**
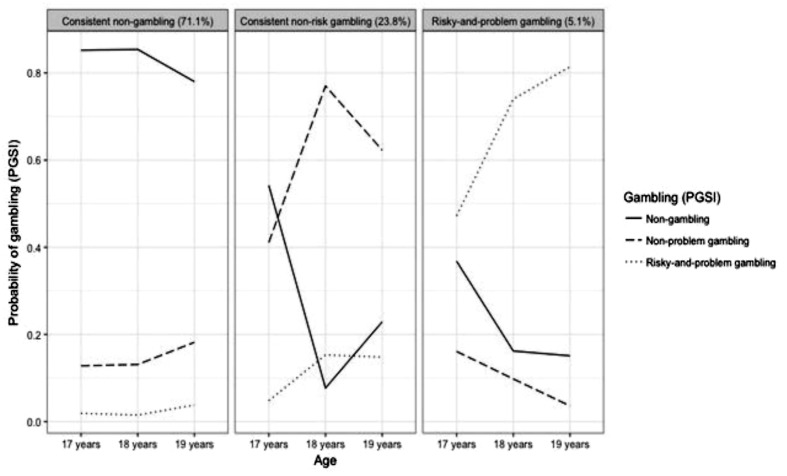
**Within-class probability of gambling (PGSI) status over repeated measurements**.

The three-class solution had the following patterns of gambling behavior. The first class, categorized “consistent non-gambling” (71.1%), comprised individuals abstaining from gambling at all measurements. The second class, labeled as “consistent non-risk gambling” (23.8%), consisted of age 17 non-gamblers participating in gambling activities without problems at ages 18 and 19. The third class, termed “risky-and-problem gambling” (5.1%), consisted of age 17 non-gamblers or gamblers (of any kind) with an increasing probability of experiencing problem gambling at ages 18 and 19. See **Figure 1**.

### Age 17 Correlates of Gambling Classes

Compared to the consistent non-gambling class, females had lower odds of belonging to the consistent non-risk gambling class. Being male, lower loneliness, higher physical aggression, higher verbal aggression, and more symptoms of depression was associated with greater odds of belonging to the risky-and-problem gambling class relative to the consistent non-gambling class. Results of the multinomial regression are presented in **Table 4**.

**Table 4:** Multinomial regression of latent gambling classes on covariates measured at age 17.

Age 17 covariate	Non-gambling vs. non-risk gambling			Non-gambling vs. risky-and-problem gambling		
		
	OR	95% CI	*p* <	OR	95% CI	*p* <
Sex^a^	0.61	0.41 to 0.91	0.016	0.11	0.05 to 0.25	0.001
Grade point average	1.28	0.96 to 1.71	0.098	1.24	0.80 to 1.91	0.330
Mother’s education	1.00	0.82 to 1.23	0.965	0.96	0.69 to 1.35	0.836
Father’s education	0.85	0.69 to 1.04	0.111	0.84	0.55 to 1.28	0.420
Loneliness^b^	0.62	0.38 to 1.01	0.056	0.30	0.12 to 0.75	0.011
Physical aggression^c^	1.31	0.98 to 1.76	0.071	1.58	1.08 to 2.30	0.017
Verbal aggression^c^	1.15	0.86 to 1.54	0.351	1.54	1.04 to 2.27	0.031
Anxiety^d^	0.96	0.90 to 1.02	0.199	1.09	0.99 to 1.19	0.095
Depression^d^	1.02	0.94 to 1.11	0.647	1.14	1.02 to 1.29	0.027


*^a^Female = 1, male = 0; ^b^Robert’s UCLA Loneliness Scale [*never* (0) to *often* (3)]; ^c^Short-Form Buss-Perry Aggression Questionnaire [*very unlike me* (0) to *very like me* (4)]; ^d^Hospital Anxiety and Depression Scale [scored from 0 (e.g., *not at all*) to 3 (e.g., *very often indeed*) with higher scores indicating higher symptoms].*

### Gambling Classes and Mental Health Endpoints at Age 19

The expected means of the mental health endpoints by latent class are shown in **Table 5**. It is evident that the consistent non-risk gambling class had the lowest level of mental health problems on all endpoints whereas the risky-and-problem gambling class had higher mental health problems on all indicators.

**Table 5:** Mental health endpoints of latent classes and latent class comparisons at age 19.

	Non-gambling		Non-risk gambling		Risky-and-problem gambling		Non-gambling vs. non-risk		Non-gambling vs. risky-and-problem		Non-risk vs. risky-and-problem	
						
Age 19 endpoint	*M*	95% CI	*M*	95% CI	*M*	95% CI	*D*^†^	95% CI	*D*^†^	95% CI	*D*^†^	95% CI
Loneliness¤	0.66	0.61 to 0.71	0.55	0.45 to 0.65	0.75	0.58 to 0.92	-0.21	-0.44 to 0.02	-0.16	-0.49 to 0.17	-0.37	-0.76 to 0.02
Physical aggression§	0.45^b^	0.39 to 0.51	0.40^a^	0.27 to 0.53	1.02^a,b^	0.65 to 1.39	-0.07	-0.3 to 0.16	-0.84	-1.4 to -0.27	-0.91	-1.54 to -0.28
Verbal aggression§	0.92^b^	0.86 to 0.98	0.89^a^	0.76 to 1.02	1.30^a,b^	0.98 to 1.62	-0.04	-0.26 to 0.18	-0.53	-1.01 to -0.05	-0.57	-1.1 to -0.04
Anxiety¤§	5.29^a^	4.99 to 5.59	4.46^a,b^	3.81 to 5.11	6.07^b^	4.87 to 7.27	-0.23	-0.45 to -0.01	-0.22	-0.57 to 0.13	-0.45	-0.86 to -0.04
Depression¤§	3.27^a^	3.01 to 3.53	2.21^a,b^	1.69 to 2.73	3.81^b^	2.77 to 4.85	-0.35	-0.57 to -0.13	-0.18	-0.55 to 0.19	-0.53	-0.95 to -0.11


*Figures sharing a superscript on the same row are significantly different (*p* < 0.05).*

*^†^Cohen’s *d*: small (0.2), medium (0.5), and large (0.8). ¤Robert’s UCLA Loneliness Scale [*never* (0) to *often* (3)]; §Short-Form Buss-Perry Aggression Questionnaire [*very unlike me* (0) to *very like me* (4)]; ¤§Hospital Anxiety and Depression Scale [scored from 0 (e.g., *not at all*) to 3 (e.g., *very often indeed*) with higher scores indicating higher symptoms].*

Additionally, compared to the consistent non-risk gambling class, the consistent non-gambling class had significantly higher anxiety and depression. In comparison to the consistent non-gambling class, the risky-and-problem gambling class had significantly higher physical and verbal aggression. Further, in comparison to the consistent non-risk gambling class, the risky-and-problem gambling class had significantly higher physical and verbal aggression, anxiety, and depression but not loneliness. Effect sizes (Cohen’s *d*; Cohen, 1988) ranged from small to large. See **Table 5**.

## Discussion

The present study longitudinally examined the association and patterns of change between mental health factors and gambling behavior in the transition from adolescence to emerging adulthood.

### Gambling Prevalence and Classes

Our finding that the highest prevalence of non-gambling is at age 17 is explainable by the illegality of gambling participation at age 17 in Norway. Similarly, our finding that age 18 has the highest prevalence of problem gambling is understandable as gambling participation in the Norwegian jurisdiction is legal at age 18. It is inferable that the novel opportunity to engage in gambling without legal restrictions at age 18 positively reinforces gambling participation at this age thereby exposing ‘novel’ or ‘first age gamblers’ to the possibility of developing gambling problems. Consistent with this phenomenon, low risk and moderate risk gambling showed increasing and marginally increasing prevalences respectively from ages 18 to 19, although the prevalence of problem gambling decreased at age 19. Operantly, the latter finding may indicate participants’ learning from their gambling problems (Shaffer and Martin, 2011). However, the above prevalence patterns should be interpreted with caution due to the low numbers in the risky-and-problem gambling category as well as the attrition rate across the waves.

Three classes of gambling behavior were identified using latent class analysis: consistent non-gambling, consistent non-risk gambling, and risky-and-problem gambling. Previous categorizations include social, at-risk, and pathological (Gupta and Derevensky, 1998) or probable pathological (Faregh and Derevensky, 2011) gamblers. Additional derived classes are low risk, at risk, problem, and pathological gamblers (Xian et al., 2008), non-problem and problem gamblers (Hong et al., 2009), as well as non-problem, preoccupied chaser, and antisocial impulsivist gamblers (McBride et al., 2010). Other classifications are non-problem, moderate problem, and pervasive problem gamblers (Carragher and McWilliams, 2011), as well as low-risk, at-risk chasing, at-risk negative consequences, and problem gambling (Kong et al., 2014). The classes identified in the present investigation are dissimilar to classes identified in previous studies especially regarding our inclusion of the consistent non-gambling class.

Several factors may account for the dissimilarities. Previous studies examined only gambling samples whereas we included non-gambling participants. Differences in study samples may also account for the differing classes as studies of adolescent (Gupta and Derevensky, 1998; Faregh and Derevensky, 2011; Kong et al., 2014) and adult (Xian et al., 2008; Hong et al., 2009; McBride et al., 2010; Carragher and McWilliams, 2011) gamblers have produced differing classes. The use of differing methods (e.g., cross-sectional vs. longitudinal) and instruments for the assessment of gambling problems, as well as differences in input categories of gambling behavior for LCAs may also account for the differences in identified classes.

### Age 17 Demographic and Mental Health Factors as Predictors of Gambling Classes

Our finding that being female is associated with lower odds of belonging to the consistent non-risk gambling class is in line with the indication from longitudinal research that the prevalence of gambling participation and disorder is higher among males than females, and that being female is associated with lower odds of experiencing gambling problems in young adulthood (Scholes-Balog et al., 2014). Similarly, the present finding that being male is associated with greater odds of belonging to the risky-and-problem gambling class supports longitudinal evidence showing that being male is a risk factor for problem gambling (Kong et al., 2014; Carbonneau et al., 2015; Pallesen et al., 2016).

Cross-sectional studies have linked loneliness to an increased risk of problem gambling (McQuade and Gill, 2012; Botterill et al., 2015; Hing et al., 2015; Brunborg et al., 2016). It is therefore surprising that loneliness at 17 years of age was associated with lower odds of belonging to the risky-and-problem gambling class in the present study. Social norms (e.g., gambling approval by family and peers, and association with family and peers with a history of gambling problems) have been associated with increased gambling involvement and problems among adolescents (Hanss et al., 2015). Additionally, social gambling (e.g., electronic gaming machines and cards) may exacerbate the risk of experiencing problem gambling and related syndromes (Goudriaan et al., 2009). Hence, it is plausible that loneliness may be protective of the social norms that facilitate or reinforce gambling (Hanss et al., 2015) as well as social gambling behavior (Quinlan et al., 2014) which is etiologically associated with problem gambling and co-occurring syndromes.

The present finding is in line with results from a cross-sectional study of gaming machine users showing that loneliness is not significantly associated with symptoms of pathological gambling (Ohtsuka et al., 1997). To our knowledge, the present study is the first to longitudinally investigate the role of loneliness in the transition to risky and problem gambling in the developmental trajectory from adolescence to emerging adulthood. Hence, our finding suggests that the cross-sectional association of loneliness with problem gambling may not be applicable longitudinally, or may have limited applicability to specific types of gambling (Goudriaan et al., 2009).

Few researchers have conducted longitudinal investigations on the association between problem gambling and aggression with results showing an enduring link between aggression and problem gambling, delineating aggression as a risk factor for problem gambling (Adachi and Willoughby, 2013; Yücel et al., 2015). Our finding that physical and verbal aggression are associated with higher odds of belonging to the risky-and-problem gambling class corroborates these results.

### Mental Health Comparison of Gambling Classes at Age 19

It is plausible that individuals with higher internalizing symptoms (e.g., high anxiety and depression) are more socially withdrawn and thus less likely to participate in social gambling activities. Our finding that the consistent non-gambling class had significantly higher symptoms of anxiety and depression than the consistent non-risk gambling class is therefore not surprising. Similarly, this finding supports the notion that moderate gambling participation may provide joy and entertainment and as such relieve symptoms of anxiety and depression. However, it should be emphasized that fixation for the attainment of these benefits may be associated with greater odds of progress to problem gambling (Wickwire et al., 2007). Also, from an addiction perspective, the present finding is similar to evidence that abstinence compared to non-abstinence from alcohol consumption is associated with greater odds of presenting anxiety and depression symptoms (Skogen et al., 2009).

Moreover, our finding that the risky-and-problem gambling class had significantly higher physical and verbal aggression than the consistent non-gambling and non-risk gambling classes is in line with results obtained in previous longitudinal studies (Adachi and Willoughby, 2013; Yücel et al., 2015). Additionally, our findings that higher symptoms of anxiety and depression were associated with the risky-and-problem gambling class supports previous findings indicating that anxiety (Ste-Marie et al., 2006; Wanner et al., 2006; Hanss et al., 2015) and depression (Beaudoin and Cox, 1999; Dussault et al., 2011; Lee et al., 2011; Chinneck et al., 2016) co-occur with or are significant risk factors for progression to problem gambling.

Consistent with the self-medication model (Khantzian, 1985), a plausible explanation for this phenomenon is that individuals engage in excessive gambling as a means of dealing with the debilitating effects of anxiety and depressive syndromes and strengthening feelings of belongingness (Beaudoin and Cox, 1999; Ste-Marie et al., 2006; Nower and Blaszczynski, 2010; Hanss et al., 2015). Moreover, our finding that the risky-and-problem gambling class had the highest physical and verbal aggression, anxiety, and depression at age 19 is in line with evidence indicating the comorbidity of gambling problems and mental health syndromes (Johansson et al., 2009; Shead et al., 2010; Chou and Afifi, 2011; Hing et al., 2015; Chinneck et al., 2016).

The present findings shed more light on the role of problem gambling in the developmental pathway to mental health syndromes. Altogether, our findings corroborate suggestion by the self-medication model of addiction (Khantzian, 1985) that individuals engage in excessive gambling in order to self-medicate or experience relief from mental health syndromes. The present findings are also explainable by the social ecology of youth culture where involvement in one problem behavior such as gambling problems is often linked with involvement in other problem behaviors and syndromes (Jessor and Jessor, 1977; Khantzian, 1985; Gupta and Derevensky, 2000; Steinberg and Morris, 2001; Wanner et al., 2006; Cheung, 2014). Further, evidence of strong associations and underlying syndromes among various addictions (Griffiths, 2005; Villella et al., 2011; Andreassen et al., 2013) may explain the present findings.

### Implications of Findings

Overall, the present findings underscore the need for recognizing externalizing factors such as physical and verbal aggression as well as internalizing factors such as anxiety and depression in the development of youth-targeted gambling interventions. Our findings also highlight the need for screening for the identified factors in the provision of treatment services for youth and individuals experiencing gambling problems. It can also be inferred from the present findings that the transition from adolescence to emerging adulthood represents an important developmental stage for ameliorative interventions targeting the risk factors identified in the present study.

### Strengths, Limitations, and Directions for Future Research

As far as we are aware, the present study is the first attempt to longitudinally investigate the associations and patterns of change between mental health factors and gambling behavior in the transition from adolescence to emerging adulthood. Relatedly, our study examines both externalizing (e.g., physical and verbal aggression) and internalizing (e.g., anxiety and depression) risk factors. As noted previously, the developmental period spanning adolescence and emerging adulthood is characterized by many biological and psychological changes (Arnett, 2004, 2007). Particularly, this period is characterized by major social change and experiences for Norwegian youth as they, for instance, can *legally* drink alcohol, drive vehicles, vote in national elections, and gamble. Hence, another noteworthy strength of our study is that it has contextually been conducted in a critical developmental stage for Norwegian youth (Arnett, 2014). Similarly, as the legal gambling age in Norway is 18 years, our longitudinal assessment of participants at 17–19 years allows for inferences regarding the class of involvement in underage or illegal gambling at age 17 to legal gambling at age 18, and a year’s span of (potential) legal gambling experience at 19 years.

Our use of latent class analysis in the identification of classes or subtypes of gambling behavior is another strength of the present study. This method builds on traditional categorization of gambling behavior. Moreover, the present study is among the first large-scale longitudinal studies of the etiological roles of aggression (Adachi and Willoughby, 2013), anxiety (Edgerton et al., 2015; Yücel et al., 2015), and depression (Chinneck et al., 2016) in the transition to problem gambling during adolescence and emerging adulthood. Additionally, to our knowledge, the present study is the first to longitudinally investigate the role of loneliness in the transition to problem gambling. Similar longitudinal studies are recommended. Particularly, longitudinal investigations are needed to further highlight the role of loneliness and participation in various types of gambling in the development of gambling problems.

Nonetheless, some limitations ought to be noted in the interpretation of our findings. First, data was obtained using self-reports that are sometimes affected by false positive responses and exaggerated estimates. Future studies may, if possible, complement self-reported data with data from other sources such as parents or guardians and peers. Moreover, although missingness was at random (MAR), the strong association of males with sample attrition should be taken into consideration when interpreting the results of the present study. Relatedly, sample attrition in the present study underlines the need for caution in comparative analysis and inferences about the pattern of gambling prevalence.

Also, in line with the original versions of the instruments, the time frame for the loneliness (RULS-8) and aggression (BPAQ-SF) responses were not indicated for each wave. It is therefore possible that responses to each wave reflect lifetime experiences. In addition, conclusions with respect to depression and anxiety may depend on which aspects of the constructs are assessed. As such, it should be noted that the HADS exclusively includes non-vegetative symptoms of depression and anxiety. The low prevalence of problem gambling in the present study should also be taken into consideration when making statistical inferences about our findings. Further, future longitudinal studies including biological markers, psychological factors such as impulsivity and substance use, gambling variables such as number of days gambled, motivations, and amount wagered may present further useful evidence on the risk factors and pathways toward gambling problems.

Although useful for investigations comprising both adolescent and adult samples, criticisms of the PGSI such as the poor discriminant validity of the low risk and moderate risk categories should be taken into consideration (Currie et al., 2013). Additionally, Cronbach’s alpha values for the verbal aggression subscale were relatively low (Nunnally, 1978). However, they were higher than the 0.60 cut-off score recommendation for short scales (Loewenthal, 2001) and therefore deemed acceptable. Further, given that our sample was representative of the Norwegian population, it comprised predominantly Caucasian individuals. Replications of the present study among ethnic minorities as well as other cultural and geographic settings are needed to throw further light on the present findings.

## Conclusion

The present population-based longitudinal investigation elucidates the significance of being male, aggression, and depression in the etiology of gambling behavior and problems in the transition from adolescence to emerging adulthood. Our findings also highlight the etiological role of gambling behavior and problems in the developmental trajectories of aggression, anxiety, and depression in the transition from adolescence to emerging adulthood. Our results denote the importance of recognizing these factors in designing targeted preventive and therapeutic interventions. Further longitudinal research is needed to identify other associated factors.

## Author Contributions

SP designed and obtained funding for the study. DS, DH, and RM managed the data collection. DS and TT conducted the statistical analyses. All authors contributed to the writing process and approved the final manuscript.

## Conflict of Interest Statement

The authors declare that the research was conducted in the absence of any commercial or financial relationships that could be construed as a potential conflict of interest.
